# Minimizing Specific Energy Consumption of Electrochemical Hydrogen Compressor at Various Operating Conditions Using Pseudo-2D Model Simulation

**DOI:** 10.3390/membranes12121214

**Published:** 2022-12-01

**Authors:** Changhyun Kim, Myungkeun Gong, Jaewon Lee, Youngseung Na

**Affiliations:** Department of Mechanical and Information Engineering, University of Seoul, Seoul 02504, Republic of Korea

**Keywords:** pseudo-two-dimensional model, electroosmotic drag, hydrogen crossover, specific energy consumption

## Abstract

With the increased usage of hydrocarbon-based fossil fuels, air pollution and global warming have accelerated. To solve this problem, renewable energy, such as hydrogen technology, has gained global attention. Hydrogen has a low volumetric density and thus requires compression technologies at high pressures to reduce storage and transportation costs. Techniques for compressing hydrogen include using mechanical and electrochemical hydrogen compressors. Mechanical compressors require higher specific energy consumption than electrochemical hydrogen compressors. Here, we used an electrochemical hydrogen compressor as a pseudo-two-dimensional model focused on electroosmotic drag, water back-diffusion, and hydrogen crossover flux at various temperatures, polymer electrolyte membrane thicknesses, and relative humidity conditions. To date, there have been few studies based on various operating conditions to find the optimal conditions. This study was conducted to determine the optimal parameters under various operating conditions. A numerical analysis demonstrated that the specific energy consumption was low in a specific current density section when the temperature was decreased. At the above-mentioned current density, the specific energy consumption decreased as the temperature increased. The polymer electrolyte membrane thickness yielded similar results. However, according to the relative humidity, it was confirmed that the higher the relative humidity, the lower the specific energy consumption in all of the current density sections. Therefore, when comparing temperatures of 30 °C and 80 °C at 145 A/$m^{2}$, operating at 30 °C reduces the specific energy consumption by 12.12%. At 3000 A/$m^{2}$ and 80 °C, the specific energy consumption is reduced by 11.7% compared to operating at 30 °C. Using N117 compared to N211 at 610 A/$m^{2}$ for polymer electrolyte membranes can reduce specific energy consumption by 10.4%. Using N211 in the 1500 A/$m^{2}$ condition reduces the specific energy demand by 9.6% compared to N117.

## 1. Introduction

With the increase in the global energy demand owing to industrial development and population growth, the use of hydrocarbon-based fossil fuels has increased, further accelerating air pollution and global warming. To solve this problem, renewable energy resources that do not emit carbon have gained attention; among them, technology that uses hydrogen has received greater attention worldwide.

Because hydrogen has a high gravimetric density, fuel cell vehicles can travel 550 km using 5 kg of hydrogen. Nevertheless, hydrogen has a very low volumetric density [1,2]. To reduce the storage and transportation costs, it is necessary to increase the volumetric density by compressing the hydrogen gas at high pressure, liquefying it, or storing it in a metal hydride [3].

Although the mechanical compressor was developed using the most advanced technological process to increase the volumetric density of hydrogen, there were problems regarding its durability owing to the moving parts of the compressor. Moreover, there are disadvantages, such as hydrogen contamination by lubricants [4].

In addition to using a mechanical compressor, electrochemical compression technology was used to compress hydrogen. Compared to mechanical compressors, electrochemical hydrogen compressors have the following advantages: an electrochemical hydrogen compressor is noiseless, has no moving parts, and can produce high-purity hydrogen [5].

Various types of electrochemical compressors include polymer electrolyte membrane water electrolysis (PEMWE) [6] (uses water), a solid acid electrochemical cell (SAEC) [7] (uses ammonia), and an electrochemical hydrogen compressor (EHC) [8] (uses hydrogen).

**Figure 1:**
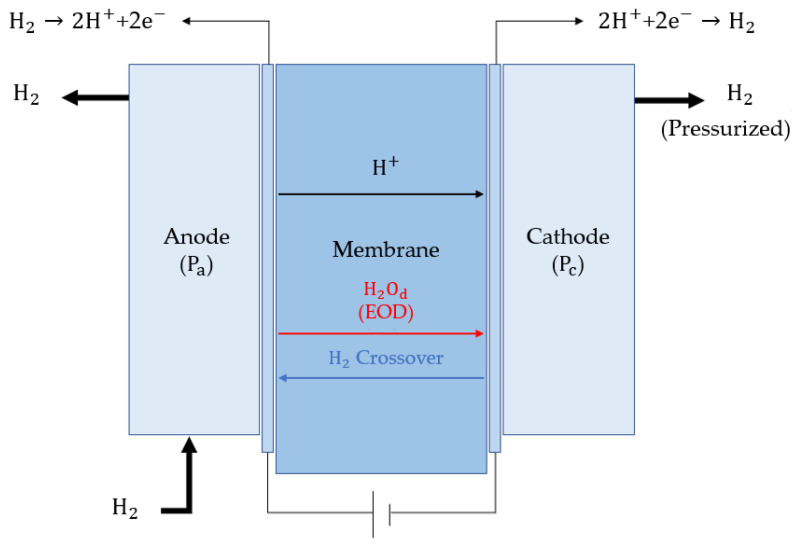
Schematic of an electrochemical hydrogen compressor.

A schematic diagram of an electrochemical hydrogen compressor with a proton exchange membrane is depicted in Figure 1. The supplied hydrogen separates into two protons and electrons in the anode’s catalyst layer (CL). The protons flow through the polymer electrolyte membrane to the CL of the cathode. The electron is transferred to the cathode via an external conductor and recombined with a proton in the CL of the cathode to form high-pressure hydrogen. The reaction between the anode and cathode is represented using Equations (1) and (2).

(1) $$\mathrm{Anode}:\;H_{2}\to 2H^{+}+2e^{-}$$

(2) $$\mathrm{Cathode}:\;2H^{+}+2e^{-}\to H_{2}$$

The disadvantage of an electrochemical hydrogen compressor is that hydrogen crossover occurs under high pressure at the cathode and moves back to the anode. Subsequently, hydrogen is discharged to the anode outlet or involved in the electrochemical reaction, further reducing the hydrogen production efficiency [9].

The HyET’s electrochemical hydrogen compressor is isothermal and single-stage, thus reducing the energy requirement by up to $3\;\mathrm{kWh}/{\mathrm{kg}}_{H_{2}}$, whereas the mechanical compressor has an additional 20% frictional energy requirement for seals and valves, adding about $6\;\mathrm{kWh}/{\mathrm{kg}}_{H_{2}}$ when compressing 1 to 40 MPa [10]. Typically, reciprocating hydrogen compressors have an average efficiency of around 45%, while electrochemical hydrogen compressors have 60% [11].

G. Sdanghi et al. analyzed the performance of an electrochemical hydrogen compressor based on the water transport mechanism [12]. They observed that when the relative humidity was below 30%, the hydration of the polymer electrolyte membrane was nonuniform, further resulting in nonuniform ionic conductivity. As a result, although the performance degraded, the focus was placed on water transport and not on hydrogen crossover. Yan Ming Hao et al. installed a humidifier at the cathode of an electrochemical hydrogen pump and conducted a study based on the operating temperature [13]. As the temperature increased, the membrane resistance decreased. Nevertheless, the performance according to the electrolyte membrane thickness was not analyzed.

Ashish Chouhan et al. analyzed the hydrogen crossover phenomenon under compressed cathode conditions up to 150 bar at low voltages [14]. They experimentally measured and formulated the hydrogen crossover rate, excluding water diffusion. R. Strobel et al. confirmed that although the electrochemical hydrogen compressor was very efficient at cathode pressures of up to 54 bar, the analysis of the amount of specific energy consumption was not performed [15]. S.A. Grigoriev et al. observed the electrochemical hydrogen compressor’s performance according to the current density and temperature [16]. It was concluded that energy was required while pressurizing from 1 to 48 bar, and the study was limited to Nafion 117. Cristina Casati et al. analyzed the performance of an electrochemical hydrogen compressor according to the applied voltage and temperature, but they did not proceed to study relative humidity [17]. J.L. Pineda-Delgado et al. evaluated the electrochemical hydrogen compressor in terms of galvanic and potentiostatic modes. They reported that the back diffusion of hydrogen does not impose a significant limitation at a pressure below 50 bar because it behaves linearly up to this pressure. The results were reported at various current densities, voltages, and cathode pressures, but temperature, electrolyte membrane thickness, and relative humidity were not considered [18].

S. Toghyani et al. analyzed the energy efficiency according to the temperature, gas diffusion layer (GDL) thickness, and cathode pressure of an electrochemical hydrogen compressor using the commercial software ANSYS Fluent [19]. Nevertheless, they did not consider the hydrogen crossover rate or water back-diffusion. Maria Nordio et al. modeled a one-dimensional electrochemical hydrogen compressor and conducted experiments and modeling tests according to changes in the hydrogen concentration, flow rate, and temperature [20]. Min Soo Kim et al. observed through experiments and simulations that the higher the temperature, the higher the voltage generated when the pressure ratio was 4.5 or higher. The parameters were the current density, operating temperature, and inlet pressure, but the electrolyte membrane thickness and relative humidity were not considered [21].

In the previous literature, few studies have conducted analyses by considering the hydrogen crossover flux and water back-diffusion. In addition, few studies have been conducted to measure the specific energy consumption and optimize the parameters by performing a numerical analysis of the electrochemical hydrogen compressor depending on the operating conditions. This study focused on the electroosmotic drag, water back-diffusion, and hydrogen crossover flux. The specific energy demand was analyzed with various temperatures, electrolyte membrane thicknesses, and relative humidity conditions. Through this study, the specific energy demand could be minimized by selecting the optimal conditions according to the operating range.

## 2. Mathematical Model

**Figure 2:**
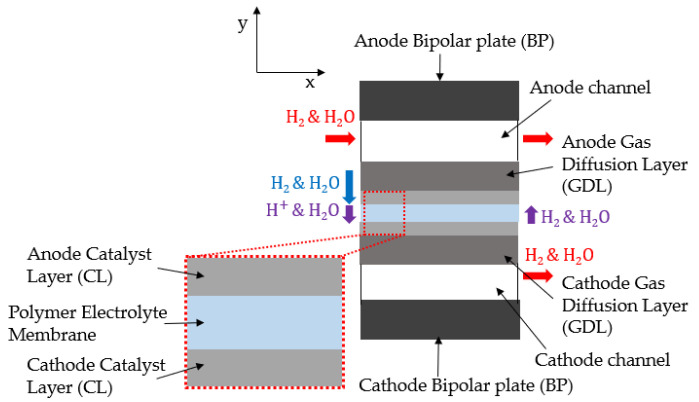
Pseudo-two-dimensional model of the electrochemical hydrogen compressor.

To proceed with the numerical analysis of the water back-diffusion and hydrogen crossover of the electrochemical hydrogen compressor, pseudo-two-dimensional (2D) modeling was performed using MATLAB 2022a. Figure 2 depicts the schematic diagram of the electrochemical hydrogen compressor of the pseudo-2D model according to the channel length, thickness of the GDL, and membrane.

The x-direction refers to the channel length, which further represents the hydrogen and water vapor flows. The y-direction refers to the GDL thickness, where hydrogen and water vapor get diffused. The electrochemical reaction was modeled in the x-direction of the catalyst. Hydrogen crossover and water back-diffusion were modeled in terms of the thickness of the polymer electrolyte membrane. The hydrogen supplied in the x-direction diffused in the y-direction and decreased from the channel inlet to the outlet. An electrochemical reaction generated and pressurized the diffused hydrogen in the y-direction at the cathode. Water vapor was also transmitted from the anode to the cathode through the polymer electrolyte membrane. When pressure was applied to the cathode, a certain amount of hydrogen passed through the membrane, and water diffused from the cathode to the anode due to the concentration difference of the electrolyte membrane. The physical properties of the model are listed in Table 1.

### 2.1. Mass Balance

**Figure 3:**
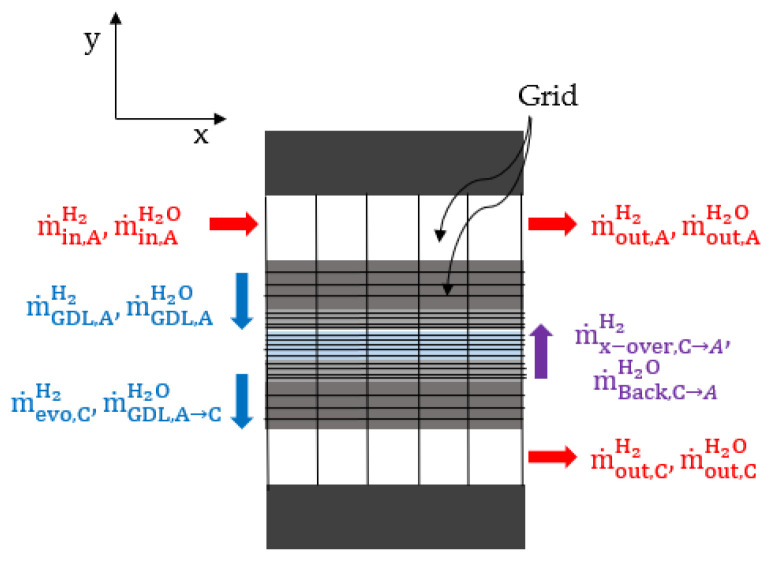
Schematic diagram of the mass balance applied to the anode and cathode.

Hydrogen and water vapor were supplied to the anode inlet; they partially diffused into the GDL, and the remaining portion was discharged through the outlet. At the cathode, hydrogen evolved from the CL, and water vapor from the anode was discharged through the outlet. Hydrogen crossover occurred at the anode as the cathode was pressurized, and with the increasing hydration of the cathode, water back-diffusion occurred in the anode owing to the concentration difference. Figure 3 depicts a schematic diagram of the mass balance. The mass balance equations can be expressed as (3)–(6).

(3) $$\mathrm{Anode}\;\mathrm{hydrogen}\;\mathrm{mass}\;\mathrm{balance}:\;{\dot{m}}_{\mathrm{in},A}^{H_{2}}-{\dot{m}}_{\mathrm{GDL},A}^{H_{2}}+{\;\dot{m}}_{x-\mathrm{over},C\to A}^{H_{2}}={\dot{m}}_{\mathrm{out},A}^{H_{2}}$$

(4) $$\mathrm{Anode}\;\mathrm{water}\;\mathrm{vapor}\;\mathrm{mass}\;\mathrm{balance}:\;{\dot{m}}_{\mathrm{in},A}^{H_{2}O}-{\dot{m}}_{\mathrm{GDL},A}^{H_{2}O}+{\;\dot{m}}_{\mathrm{Back},C\to A}^{H_{2}O}={\;\dot{m}}_{\mathrm{out},A}^{H_{2}O}$$

(5) $$\mathrm{Cathode}\;\mathrm{hydrogen}\;\mathrm{mass}\;\mathrm{balance}:\;{\dot{m}}_{\mathrm{evo},C}^{H_{2}}-{\dot{m}}_{x-\mathrm{over},C\to A}^{H_{2}}={\;\dot{m}}_{\mathrm{out},C}^{H_{2}}$$

(6) $$\mathrm{Cathode}\;\mathrm{water}\;\mathrm{vapor}\;\mathrm{mass}\;\mathrm{balance}:\;{\;\dot{m}}_{\mathrm{GDL},A\to C}^{H_{2}O}-{\dot{m}}_{\mathrm{Back},C\to A}^{H_{2}O}={\;\dot{m}}_{\mathrm{out},C}^{H_{2}O}$$

### 2.2. Gas Diffusion Layer Modeling

In the GDL, the diffusion of hydrogen and water vapor occurred, which can be calculated using the Maxwell–Stefan Equation (7) [26].

(7) $$\frac{{\mathrm{dx}}_{\mathrm{sa}}}{\mathrm{dy}}=\mathrm{RT}\sum_{\mathrm{sb}}\frac{x_{\mathrm{sa}}{\dot{N}}_{\mathrm{sb}}-x_{\mathrm{sb}}{\dot{N}}_{\mathrm{sa}}}{{\mathrm{PD}}_{\mathrm{sa},\mathrm{sb}}}$$

Hydrogen and water vapor are diffusivity processes; therefore, the binary diffusivity should be calculated using Equation (8) [27].

(8) $$D_{\mathrm{sa},\mathrm{sb}}=\frac{0.164}{P}{\left(\frac{T}{303}\right)}^{\frac{3}{2}}\varepsilon_{\mathrm{GDL}}^{\frac{3}{2}}$$

### 2.3. Overpotential Modeling

The voltage applied to the electrochemical hydrogen compressor can be expressed as the sum of the Nernst potential, activation losses, and ohmic losses, as shown in Equation (9) [20].

(9) $$E_{\mathrm{Total}}=\left(E_{\mathrm{Nernst},A}+E_{\mathrm{act},A}\right)-\left(E_{\mathrm{Nernst},C}+E_{\mathrm{act},C}\right)+E_{\mathrm{ohmic}}$$

The current density generated through the electrochemical reaction was calculated using the Butler–Volmer equation, as shown in Equations (10) and (11) [20].

(10) $$i={\mathrm{FK}}_{o}[a_{H_{2},\;A}^{0.5}e^{\frac{\alpha_{H2}F}{\mathrm{RT}}\left(E_{\mathrm{Nernst},A}+E_{\mathrm{act},A}\right)}-e^{-\frac{\alpha_{H2}F}{\mathrm{RT}}\left(E_{\mathrm{Nernst},A}+E_{\mathrm{act},A}\right)}]$$

(11) $$-\;i={\mathrm{FK}}_{r}[a_{H_{2},C}^{0.5}e^{\frac{\alpha_{H2}F}{\mathrm{RT}}\left(E_{\mathrm{Nernst},C}+E_{\mathrm{act},C}\right)}-e^{-\frac{\alpha_{H2}F}{\mathrm{RT}}\left(E_{\mathrm{Nernst},C}+E_{\mathrm{act},C}\right)}]$$

To separate the Nernst potential and activation losses from Equation (4), they can be expressed as Equations (12) and (13) [20].

(12) $$E_{\mathrm{Nernst},A}=\frac{\mathrm{RT}}{n_{e}F}\ln(\frac{1}{\frac{x_{s}p_{A}}{p_{\mathrm{amb}}}})$$

(13) $$E_{\mathrm{Nernst},C}=-\frac{\mathrm{RT}}{n_{e}F}\ln(\frac{p_{C}}{p_{\mathrm{amb}}})$$

To obtain ohmic losses, the resistance of the polymer electrolyte membrane should be calculated using Equation (14), which can be expressed as a function of the water content (λ) and ionic conductivity (σ) [22]


(14) $${\mathrm{ASR}}_{\mathrm{mem}}=\int_{0}^{t_{\mathrm{mem}}}\frac{\mathrm{dy}}{\sigma \left(\lambda \right)}$$

The ionic conductivity can be calculated using Equation (15) as a function of the water content and temperature [22].

(15) $$\sigma_{\mathrm{mem}}=\left(0.5193\lambda -0.326\right)e^{1268\left(\frac{1}{303}-\frac{1}{T}\right)}$$

As a result, ohmic losses can be expressed using Equation (16) [22]:

(16) $$E_{\mathrm{ohmic}}=i\;\times {\;\mathrm{ASR}}_{\mathrm{mem}}$$

### 2.4. Polymer Electrolyte Membrane Modeling

In the polymer electrolyte membrane, water moved from the anode to the cathode via electroosmotic drag. As the water flux increased from the anode to the cathode, the water content in the cathode increased, and back diffusion occurred owing to the difference in concentration. The electroosmotic drag and back diffusion can be expressed by Equation (17) [22].

(17) $$J_{H_{2}O}=2n_{\mathrm{drag}}^{\mathrm{SAT}}\frac{i}{n_{e}F}\frac{\lambda }{22}-\frac{\rho_{\mathrm{dry}}}{M_{\mathrm{mem}}}D_{\lambda }\frac{d\lambda }{\mathrm{dy}}$$

The hydrogen crossover, depending on the pressure difference between the anode and cathode, can be expressed using Equation (18) [28].

(18) $${\dot{n}}_{x}=\left(-2.6492+0.018\left(T-273.15\right)+0.0036\mathrm{RH}+0.5992\left(\frac{P_{C}-P_{A}}{100,000}\right)+\frac{10.84}{\ln\left(t_{\mathrm{mem}}\right)}\right)\times 10^{-9}$$

### 2.5. Validation of Modeling

**Figure 4:**
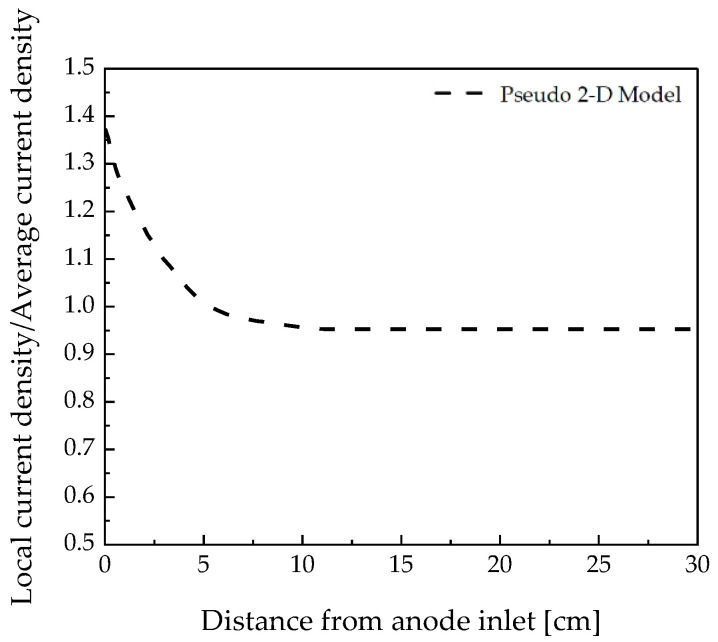
Comparison of local current density/average current density according to the anode inlet distance analyzed by the pseudo-2D model and reference model.

To verify the reliability of the pseudo-2D model used in this study, it was compared with the numerical analysis and experimental results reported by G. Sdanghi et al. [23]; the results are depicted in Figure 4. The operating conditions were set to a temperature of 60 °C, relative humidity of 90%, $P_{c}$ of 4 bar, and Nafion 117.

The pseudo-2D model could achieve similar results to the numerical analysis and experimental results of G. Sdanghi et al. within an error of 4% [23]. Therefore, additional studies were conducted under various operating conditions using the pseudo-2D model. The specific energy consumption was calculated using Equation (19) to determine the optimal conditions of the electrochemical hydrogen compressor.

(19) $$E_{\mathrm{Cons},{\mathrm{kg}}_{H_{2}}}=\frac{P_{\mathrm{Cell}}}{m_{\mathrm{Prod},H_{2}}-m_{x-\mathrm{over},H_{2}}}$$

## 3. Results and Discussion

This study analyzed the specific energy consumption depending on the operating temperature, polymer electrolyte membrane thickness, and relative humidity. Table 2 lists the operating conditions for the numerical analysis.

Relative humidity (RH)

### 3.1. Temperature Effects

**Figure 5:**
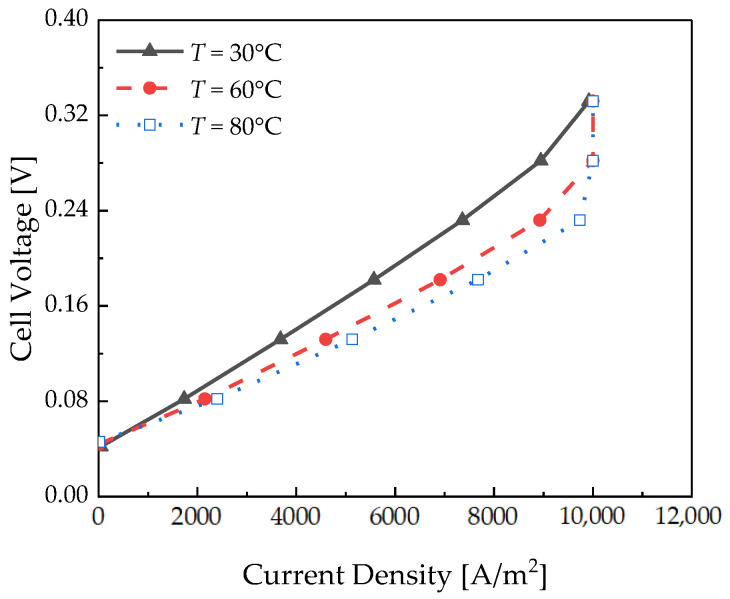
Polarization curve of the pseudo-2D electrochemical hydrogen compressor with respect to temperature.

A numerical analysis was performed under the following operating conditions: a relative humidity of 100%, Nafion 115, and $P_{c}$ of 100 bar. Figure 5 depicts the polarization curve measured with respect to temperature.

**Figure 6:**
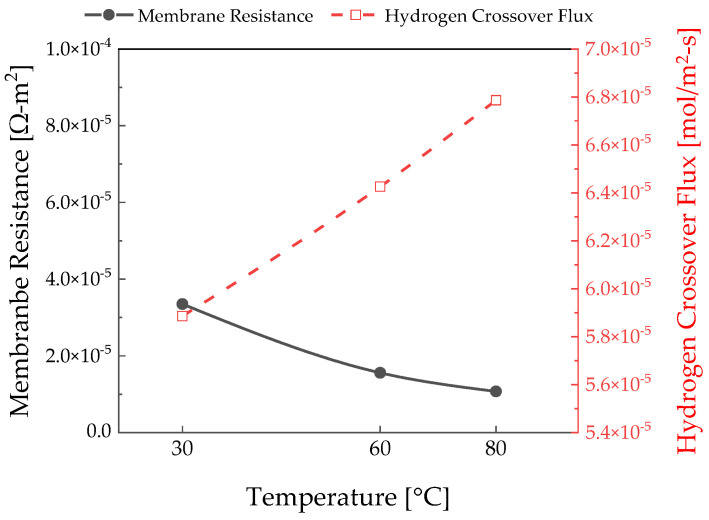
Polymer electrolyte membrane resistance and hydrogen crossover flux according to the temperature measured at 0.18 V.

The slope of the polarization curve increased as the temperature decreased. Ions slowly moved through the polymer electrolyte membrane at low temperatures; therefore, the ohmic losses increased at 0.18 V, as depicted in Figure 6. As shown in Equation (16), the ionic conductivity decreases as the temperature decreases, which further increases the membrane resistance.

**Figure 7:**
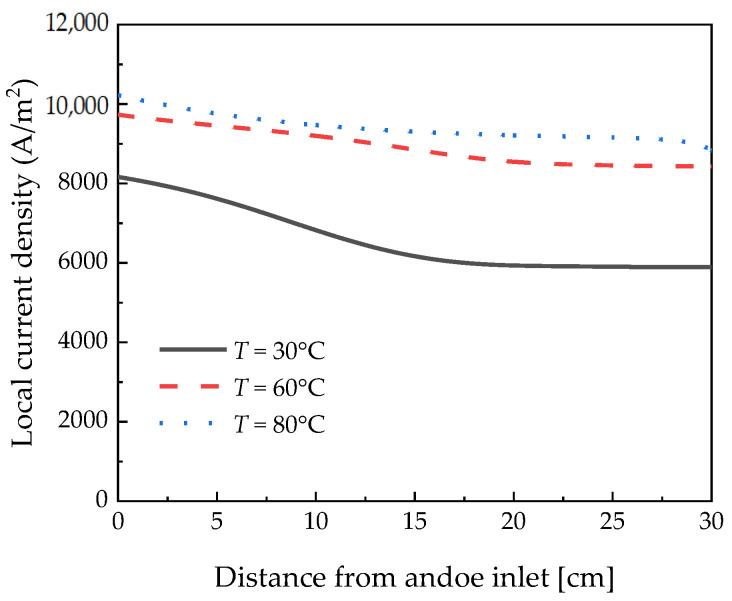
Local current density measured along the channel length according to the temperature at 0.18 V.

Consequently, it can be confirmed that the local current density decreased with a decrease in the temperature, as depicted in Figure 7. The local current density decreased up to 15 cm under all temperature conditions and then reached a steady state. It is considered that the hydration of the membrane decreases up to 15 cm under the 0.18 V condition and then converges to a steady state. Water drag to the cathode lowered the current density at the outlet. This water shortage is more severe at lower temperatures than at higher temperatures with significant water content. At 30 °C, the saturated water vapor pressure is low, so the membrane dries quickly.

**Figure 8:**
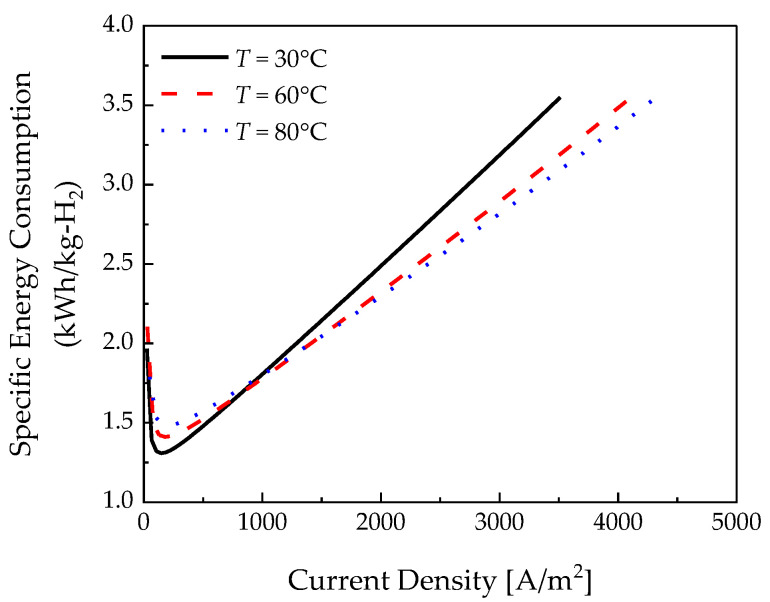
Specific energy consumption with respect to temperature.

As the temperature increases, the hydrogen crossover flux increases at 0.18 V, as depicted in Figure 6, because of the binary diffusivity in Equation (8) [28]. Figure 8 depicts the required specific energy consumption depending on the temperature. As the effect of hydrogen crossover was dominant in the low-current-density area, the specific energy consumption increased as the temperature increased. As can be seen from Equation (18), the hydrogen crossover flux increases as the temperature increases. Compared with 30 °C and 80 °C at 145 A/$m^{2}$, there is a 12.12% difference in specific energy consumption. At a high current density, a lot of hydrogen is produced, so the effect of the hydrogen crossover flux is not significant. However, in the high-current-density region, the ohmic losses increased linearly, further decreasing the specific energy consumption at higher temperatures. In the case of 3000 A/$m^{2}$, the specific energy consumption is reduced by 11.7% at 80 °C compared to 30 °C. Therefore, it became more efficient as the temperature increased when the current density was above 1000 A/$m^{2}$. The analysis of the effect of temperature shows that the resistance of the polymer electrolyte membrane had a greater effect on the specific energy consumption than the effect of the hydrogen crossover flux in a region above a specific electrical current density.

### 3.2. Membrane Thickness Effects

**Figure 9:**
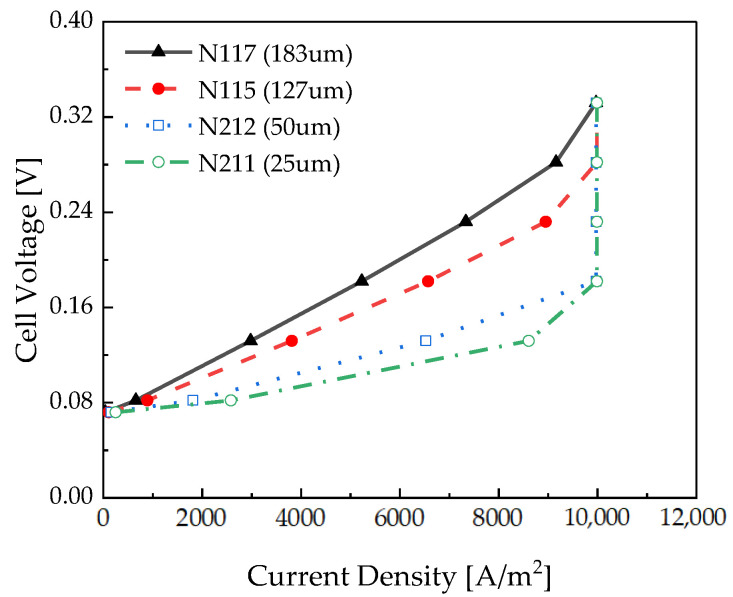
Polarization curve of the pseudo-2D electrochemical hydrogen compressor according to the polymer electrolyte membrane thickness.

The effect of thickness was analyzed using various polymer electrolyte membranes. Figure 9 depicts the polarization curve of the electrochemical hydrogen compressor as a function of the thickness of the polymer electrolyte membrane. The operating conditions were set to a temperature of 30 °C, a relative humidity of 100%, and $P_{c}$ of 100 bar. The results demonstrate that the gradient of the polarization curve increases with an increase in the polymer electrolyte membrane thickness.

**Figure 10:**
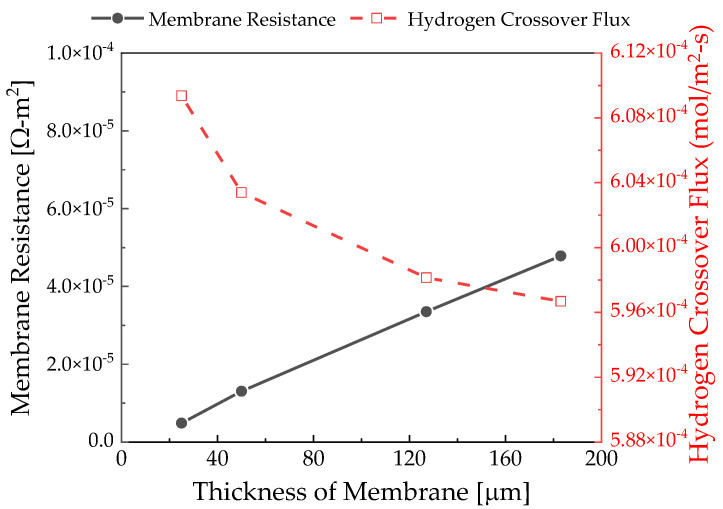
Polymer electrolyte membrane resistance and hydrogen crossover rate according to the thickness measured at 0.18 V.

As depicted in Figure 10, the membrane resistance increases because the ion migration distance increases according to the membrane thickness. The hydrogen crossover tended to increase exponentially as the membrane became thinner. As can be seen from the results of Yuan et al. [29], with membrane degradation, the hydrogen crossover did not change significantly in the case of a thick membrane but increased dramatically in the case of a thin membrane. Therefore, the hydrogen crossover rate in Equation (18) is obtained in inverse proportion to the value obtained by taking the natural logarithm of the membrane thickness. Hydrogen crossover is more sensitively affected when the film is thin.

**Figure 11:**
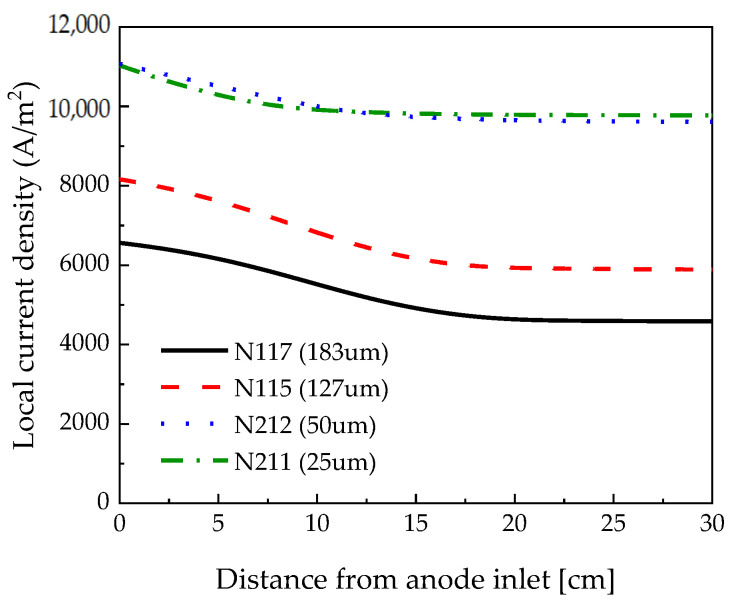
Local current density measured along the channel length according to the polymer electrolyte membrane thickness at 0.18 V.

Figure 11 depicts the local current density depending on the channel length measured at 0.18 V. Similar local current densities were observed at 25 μm and 50 μm because they were measured in the limiting current density range. However, compared to 50 μm, the local current density at 25 μm was lower near the inlet. In the case of 25 μm, the thin membrane caused more electroosmotic drag and decreased hydration near the inlet. Near the channel outlet, the local current density was slightly higher at 25 μm compared to its value at 50 μm, which is considered to be the effect of back diffusion. When the membrane thickness increased from 127 µm to 183 µm, the local current density was low because the membrane resistance increased with thick membranes. Water back-diffusion equilibrated the electroosmotic drag earlier in the thin membrane. So, the local current density of the N117 membrane was the latest to stabilize compared to the other membranes.

**Figure 12:**
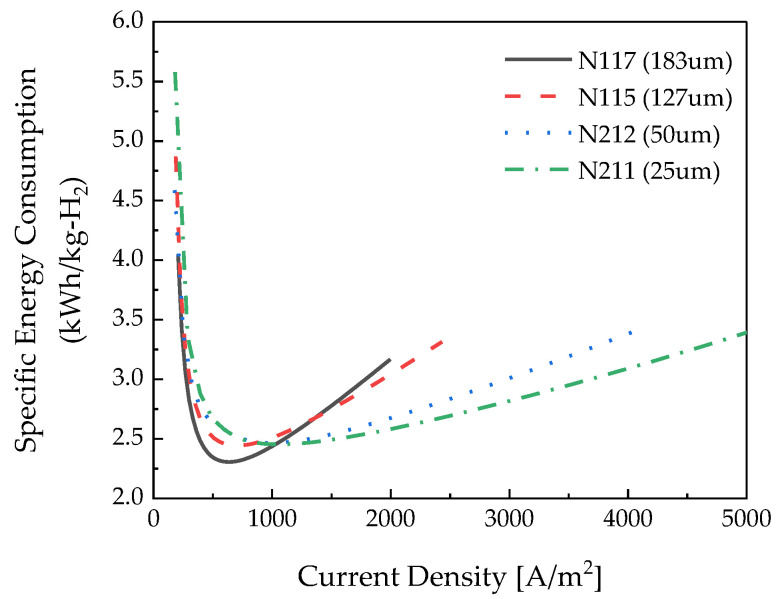
Specific energy consumption according to the polymer electrolyte membrane thickness.

Figure 12 depicts the specific energy consumption as a function of the thickness of the polymer electrolyte membrane. As the polymer electrolyte membrane thickness increased, the specific energy consumption decreased in the low-current-density section. In the low-current-density region, as depicted in Figure 9, the hydrogen crossover flux was dominant over the hydrogen produced.

In the high-current-density section, the thicker the electrolyte membrane, the higher the specific energy consumption. The effect of the membrane resistance was greater than that of the hydrogen crossover flux in the high-current-density region. Comparing the thickest membrane and the thinnest membrane at a low current density, the specific energy consumption differs by 10.4%, and at a high current density, a difference of 9.6% occurs.

It can be seen that the thicker the electrolyte membrane is, the lower the specific energy consumption in the low-current-density section is, and the thinner the membrane is, the more suitable it is to operate in the high-current-density section.

### 3.3. Relative Humidity Effects

**Figure 13:**
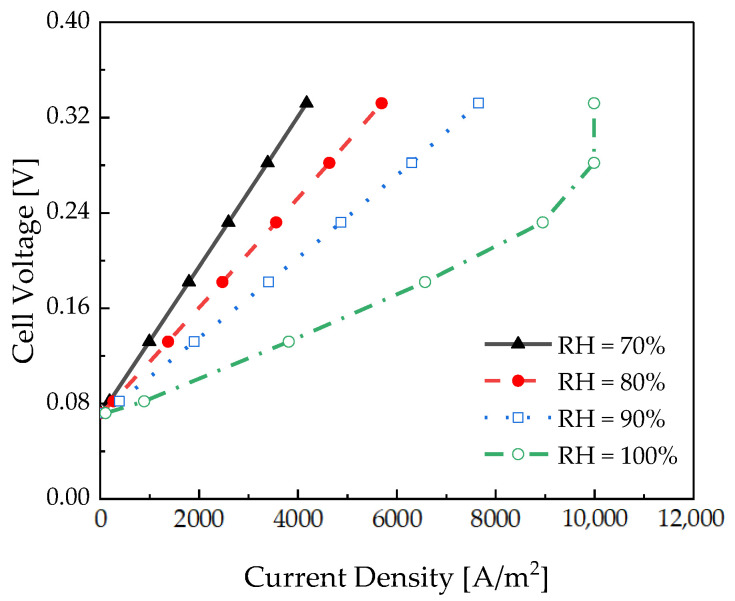
Polarization curve of the pseudo-2D electrochemical hydrogen compressor with respect to the relative humidity.

The effect of the relative humidity was analyzed using a pseudo-2D model. Figure 13 depicts the polarization curve of the electrochemical hydrogen compressor with respect to the relative humidity. The operating conditions were as follows: temperature of 30 °C, Nafion 115, and $P_{c}$ of 100 bar. The results showed that the slope of the polarization curve increased as the relative humidity decreased because the higher the relative humidity, the larger the amount of water vapor supplied to the channel, which increases the ionic conductivity of Nafion from the channel inlet.

**Figure 14:**
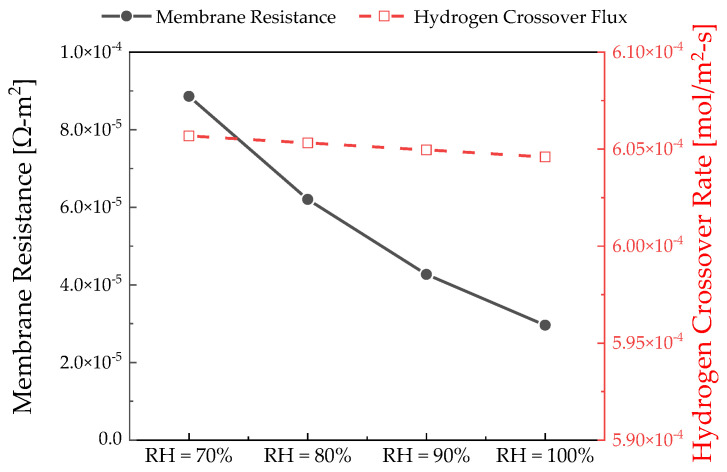
Polymer electrolyte membrane resistance and hydrogen crossover flux according to the thickness measured at 0.18 V.

As depicted in Figure 14, partially humidified water vapor was supplied, and the membrane resistance increased as the dehydration of the membrane increased. As a result, the performance of the electrochemical hydrogen compressor degraded.

**Figure 15:**
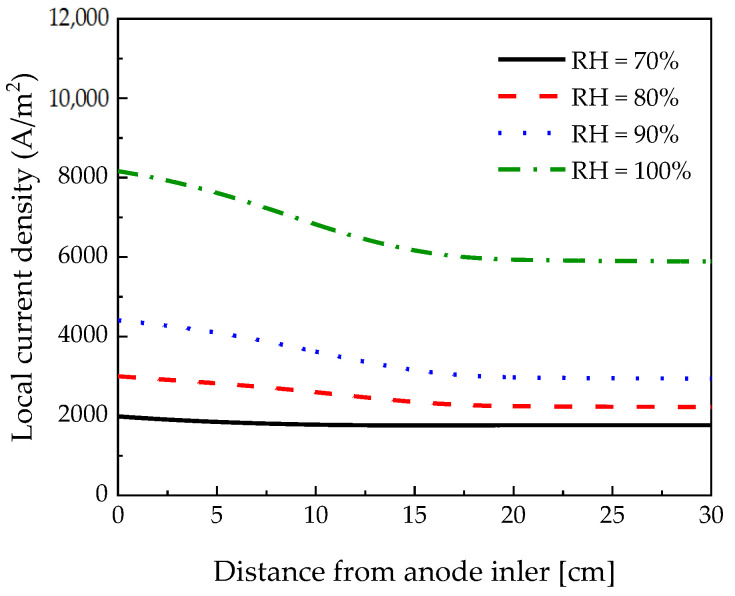
Local current density measured along the channel length according to the relative humidity at 0.18 V.

As depicted in Figure 15, the local current density decreased when the relative humidity decreased from 100% to 70%. As the relative humidity increased, the local current density near the inlet decreased rapidly because of the high water vapor activity, which improved the ionic conductivity and increased the electroosmotic drag. It can be seen that the lower the relative humidity, the less the electroosmotic drag, so the local current density is almost constant.

**Figure 16:**
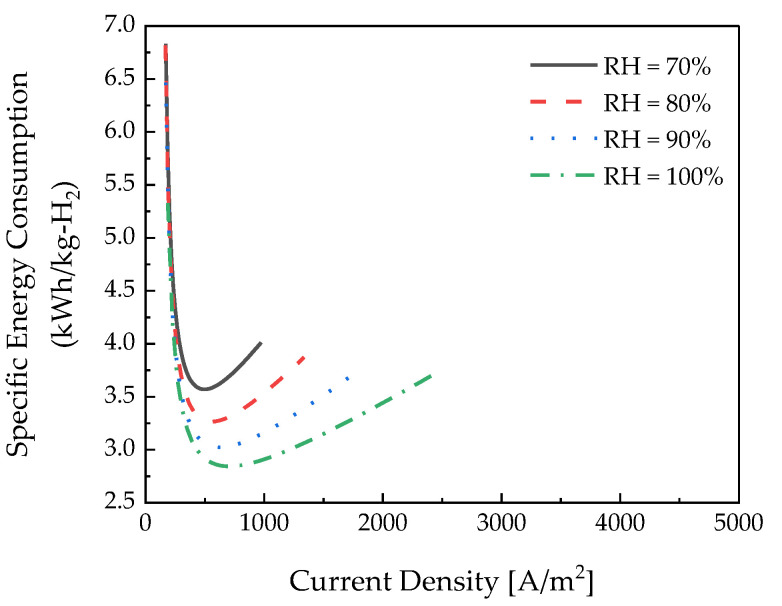
Specific energy consumption according to the polymer electrolyte membrane thickness.

Figure 16 depicts the specific energy consumption according to the relative humidity. The relative humidity was affected by the membrane resistance. Consequently, it was confirmed that the specific energy consumption decreased as the relative humidity increased.

In the case of the hydrogen crossover flux, it is evident that the amount of hydrogen transmitted from the cathode to the anode was similar according to the relative humidity. This is because the relative humidity does not significantly affect the hydrogen crossover flux, as shown in Equation (18). Therefore, it can be confirmed that the higher the relative humidity, the lower the specific energy consumption, regardless of the current density.

## 4. Conclusions

A pseudo-2D model was used to determine the optimal conditions of an electrochemical hydrogen compressor according to the temperature, polymer electrolyte membrane thickness, and relative humidity. The specific energy consumption was used as a performance indicator, and the results were analyzed in a specific current density section.

As a result of analyzing the effect of the parameters, a similar current density was measured in the low-current-density region. In this case, although the produced hydrogen was similar, the hydrogen crossover increased, so it was possible to reduce specific energy consumption by using 30 °C, N117, and a relative humidity of 100% at a current density lower than 1000 A/$m^{2}$. The hydrogen production increased in the high-current-density section, and the effect on hydrogen crossover was not dominant. On the contrary, the effect on ohmic losses increased; if the current density is above 1000 A/$m^{2}$, it must be operated at 80 °C, N211, and a relative humidity of 100% to reduce specific energy consumption.

In conclusion, we found that maintaining high relative humidity at all times was a condition for reducing specific energy consumption. Specifically, it was confirmed that the temperature and polymer electrolyte membrane thickness affected the specific energy consumption according to the current density. Therefore, the electrochemical hydrogen compressor is efficient when operated at a low temperature, thick electrolyte membrane, and at high relative humidity at a low current density. At a high current density, it is efficient to operate at a high temperature, thin electrolyte membrane, and high relative humidity. In addition, in this study, we considered a pseudo-2D model; we believe that optimization variables can be derived within a short time.

## Figures and Tables

**Table 1:** Physical parameters and geometrical properties of EHC.

Parameter	Units	Value
Faraday constant (F)	C/mol	96,485.332
Gas constant (R)	J/mol∙K	8.3144
Equivalent weight of membrane ($M_{\mathrm{mem}}$)	kg/kmol	1100 [22]
Dry density of membrane ($\rho_{\mathrm{dry}}$)	$\mathrm{kg}/m^{3}$	1970 [22]
Channel length ($l_{\mathrm{ch}}$)	mm	300 [23]
Thickness of bipolar plate ($t_{\mathrm{BP}}$)	mm	1
Through-plane electrical conductivity of bipolar plate ($\sigma_{\mathrm{BP}}$)	S/m	3.3 [24]
Thickness of gas diffusion layer ($t_{\mathrm{GDL}}$)	μm	325 [25]
Through-plane electrical conductivity of gas diffusion layer ($\sigma_{\mathrm{GDL}}$)	S/m	220 [25]
Gas diffusion layer porosity ($\varepsilon_{\mathrm{GDL}}$)	-	0.5 [25]
Polymer electrolyte membrane thickness ($t_{\mathrm{mem}}$)	μm	25, 50, 127, 183

**Table 2:** Operating conditions of the electrochemical hydrogen compressor.

Parameter	Units	Value
Operating temperature (T)	°C	30, 60, 80
Operating pressure (P)	bar	100
Relative humidity (RH)	%	100, 90, 80, 70
Flow rate	sccm	41.4

## Data Availability

Not applicable.

## Abbreviations

List of symbols	
a	Activity/-
ASR	Area-specific resistance/$Ω{\;m}^{2}$
D	Binary diffusivity/$m^{2}{\;s}^{-1}$
$D_{\lambda }$	Water diffusivity/$m^{2}{\;s}^{-1}$
E	Cell voltage/V
F	Faraday constant/${A\;s\;\mathrm{mol}}^{-1}$
i	Current density/${A\;m}^{-2}$
J	Water flux considering electroosmotic drag and back diffusion/mol ${\;s}^{-1}{\;m}^{-2}$
K	Kinetic constant/${\mathrm{mol}\;s}^{-1}{\;m}^{-2}$
$\dot{m}$	Mass flow rate/${\mathrm{kg}\;s}^{-1}$
M	Equivalent weight of membrane/${\mathrm{kg}\;\mathrm{mol}}^{-1}$
n	Number of electrons in hydrogen
${\dot{n}}_{x}$	Hydrogen crossover flux/mol ${\;s}^{-1}{\;m}^{-2}$
$\dot{N}$	Molar flux/${\mathrm{mol}\;m}^{-2}{\;s}^{-1}$
P	Pressure/bar
$P_{\mathrm{Cell}}$	Power/W
R	Ideal gas constant/${J\;\mathrm{mol}}^{-1}{\;K}^{-1}$
t	Thickness/mm
T	Temperature/K
x	Mole fraction/-
Greek letters	
α	Transfer coefficient/-
ε	Porosity/-
ρ	Density/${\mathrm{kg}\;m}^{-3}$
Subscripts and superscripts	
act	Activation losses
amb	Ambient
A	Anode
A→C	Anode to cathode
Back	Back diffusion
BP	Bipolar plate
C	Cathode
C→A	Cathode to anode
Cons	Consumption
drag	Drag coefficient
evo	Evolution
e	Electron
GDL	Gas diffusion layer
$H_{2}$	Hydrogen
$H_{2}O$	Water
in	Inlet
mem	Polymer electrolyte membrane
Nernst	Nernst potential
out	Outlet
ohmic	Ohmic losses
o	Oxidation of hydrogen
Prod	Production
r	Reduction of hydrogen
sa	Chemical species of a
sb	Chemical species of b
s	Surface
SAT	Saturation
Total	Applied voltage to the EHC
x−over	Hydrogen crossover
